# Prognostic models of multisystem inflammatory syndrome severity according to resource availability in healthcare facilities in Ukraine

**DOI:** 10.3389/fped.2026.1740828

**Published:** 2026-02-26

**Authors:** N. Y. Bodnarchuk-Sokhatska, H. A. Pavlyshyn

**Affiliations:** Department of Pediatrics No. 2, I. Horbachevsky Ternopil National Medical University, Ternopil, Ukraine

**Keywords:** ESR, interleukin-6, multisystem inflammatory syndrome in children, pediatric intensive care, prognostic model, SARS-CoV-2, severity score

## Abstract

**Background:**

Multisystem inflammatory syndrome in children (MIS-C) remains one of the most complex post-infectious conditions associated with SARS-CoV-2. In Ukraine, where the healthcare system operates under uneven resource availability due to the ongoing conflict, diagnostic and therapeutic decisions often require simplified, adaptable tools for early risk assessment. This study aims to develop MIS-C severity scoring systems based on clinical and laboratory predictors and differentiate them according to healthcare facilities resource availability.

**Methods:**

This retrospective cohort study included 51 children (aged 7 months to 17 years) with confirmed MIS-C. Patients were divided into severe (PICU+) and non-severe (PICU–) groups. MLR was used to identify independent predictors, followed by internal bootstrap validation. Two resource-adjusted models were proposed: MIS-C Severity Score Basic (MIS-C-SS-Basic) and MIS-C Severity Score Advanced (MIS-C-SS-Advanced).

**Results:**

The Advanced model included four-system involvement, hepatomegaly, and IL-6 ≥ 310 pg/mL and showed AUC = 0.936 with sensitivity 91.7% and specificity 88.9%. The Basic model included ESR ≥ 22 mm/h, hepatomegaly, and free abdominal fluid and showed AUC = 0.826 with sensitivity 87.5% and specificity 70.4%. Both models were converted into point-based scores.

**Conclusion:**

Two resource-adapted MIS-C severity scores were developed to predict PICU admission in children with MIS-C. External validation in independent cohorts is needed.

## 1 Introduction

Multisystem inflammatory syndrome in children is a challenging diagnosis not only in the medical field, but also a problem of socio-economic importance. In the context of logistical support for healthcare facilities (HCFs), the significant resources required for timely diagnostics and treatment place a substantial burden on the financial systems of medical institutions and influence state healthcare policy, in particular (1, 2).

This issue is particularly pronounced in resource-limited settings (RLS), especially in low- and middle-income countries (LMICs). Ukraine used to be classified among these countries.

Although, according to the World Bank classification, as of 2024 Ukraine belongs to the group of upper-middle-income countries (UMICs), this classification upgrade does not fully reflect the actual consequences of the ongoing war — including the destruction of infrastructure, shortage of medical personnel, disruption of medicine supply chains, and the unequal access to basic healthcare services across different regions of the country (3–7).

This discrepancy between Ukraine's formal macroeconomic status and its real clinical and organizational conditions justifies the need for the development of adaptive, resource-sensitive clinical tools — such as severity stratification scales for MIS — that are suitable for use across healthcare facilities with varying levels of resource availability.

The diagnosis of MIS-C associated with COVID-19 in LMICs is primarily limited by restricted access to key laboratory tests such as lactate, N-terminal pro–B-type natriuretic peptide (NT-proBNP), D-dimer, interleukin-6 (IL-6) etc. In particular, such a situation has been described in the Lao People's Democratic Republic, where therapeutic options and the timeliness of intervention are further constrained by the substantial cost of intravenous human immunoglobulin (IVIG) (8). As a preventive measure, adapted MIS-C management protocols were developed in Kenya, taking into account the limited access to pediatric intensive care in some regions (9). A report from Armenia indicates effective management of children with MIS in general pediatric wards without access to pediatric intensive care units (PICUs), provided that an appropriate patient pathway and a combined therapy with glucocorticoids and immunoglobulins (10). These examples highlight the need for developing an adaptive MIS-C severity stratification scale that accounts for the level of resource availability in healthcare facilities.

According to the literature, reported cases of MIS-C in Ukraine emphasize the importance of establishing a clear diagnostic algorithm for early detection, even in regional healthcare facilities with extensive laboratory and instrumental capabilities (11).

Decision-making regarding disease severity, and consequently the management strategy and patient care within healthcare facilities with both limited and expanded resources, necessitates the development of a clear protocol for healthcare personnel interaction within the institution, while simultaneously providing a unifying framework for coordination between different facilities. Accordingly, such adaptive MIS-C severity assessment scales for children, derived from clinically relevant laboratory and clinical criteria, facilitate the clinical decision-making process, optimize patient pathways, and rationalize the use of available healthcare resources, particularly in crisis situations, such as armed conflicts, or limited access to intensive care and medications. The aim of this study was to develop logistic regression–based severity assessment scales for MIS in children that integrate key clinical and laboratory predictors, and to verify their characteristics under conditions of both high and limited levels of healthcare resource availability.

## 2 Materials and methods

### 2.1 Study design

A retrospective cohort study was conducted, including 51 cases of MIS-C with post–SARS-CoV-2 infection. All registered cases involved children aged 7 months to 17 years who were hospitalized for inpatient treatment in healthcare facilities with varying levels of resource availability. The verification of MIS-C diagnosis was performed according to the CSTE/CDC diagnostic criteria (2022) (12) with exclusion of active acute infection in patients.

#### 2.1.1 Eligibility criteria

Inclusion criteria were: (1) age 0–18 years; (2) hospitalization for inpatient treatment; and (3) fulfillment of the CSTE/CDC MIS-C surveillance case definition (approved in 2022; effective January 1, 2023). Accordingly, included cases had fever and evidence of systemic inflammation (CRP ≥ 3.0 mg/dL) and demonstrated new onset manifestations in ≥2 of the following categories: cardiac, mucocutaneous, shock, gastrointestinal, and hematologic, together with laboratory evidence of SARS-CoV-2 infection or epidemiologic linkage to a confirmed COVID-19 case.

Exclusion criteria were: (1) evidence of active acute SARS-CoV-2 infection at presentation when an acute infection better explained the clinical syndrome; (2) an alternative diagnosis fully explaining the presentation and/or judged more likely than MIS-C, including bacterial sepsis or toxic shock syndrome, other acute infections, Kawasaki disease, systemic rheumatic disease (e.g., systemic juvenile idiopathic arthritis/Still's disease), macrophage activation syndrome, and (3) insufficient documentation to adjudicate the case definition.

To maintain consistency with the CSTE/CDC MIS-C surveillance case definition, respiratory and neurological findings were treated as clinical manifestations (not organ-category criteria for case ascertainment) and were operationalized as follows. Respiratory involvement was defined as the presence of any baseline respiratory symptom/sign (cough and/or dyspnea and/or pneumonia and/or сhest pain/tightness). Neurological involvement was defined as the presence of any baseline neurological manifestation (headache, аltered mental status (drowsiness/lethargy, irritability/restlessness), meningeal signs/meningism or encephalopathy). Laboratory evidence of coagulopathy was defined *a priori* as any of the following at baseline: prolonged PT and/or prolonged aPTT and/or elevated D-dimer and/or abnormal fibrinogen (based on local laboratory reference ranges).

Clinical and laboratory data were collected retrospectively from inpatient medical records. Predictors were defined from the initial clinical assessment and the earliest available laboratory and instrumental (including ultrasound) measurements obtained within the first 24–48 h of hospitalization and prior to IVIG administration. Laboratory test results were obtained at a median of 6.0 (5.0; 7.0) days from disease onset. Measurements obtained later during the hospital course were not used as predictors in model development.

#### 2.1.2 Ultrasound assessment

Abdominal ultrasound was part of the routine diagnostic work-up at admission for suspected MIS-C and was available for all included patients. Ultrasound findings were extracted retrospectively from the original radiology reports. Free abdominal fluid was coded as present when any serous fluid was reported in the peritoneal cavity at any location, including small volume (“minimal/strip-like”) effusions; when documented, the maximal fluid depth was recorded in millimeters, but for modeling we used the binary presence/absence variable.

#### 2.1.3 Definition of severe disease and outcome

Severe disease was defined as PICU admission during the MIS-C episode. Accordingly, patients were classified as PICU (+) – children who required admission to the intensive care unit during the MIS-C episode, and PICU (-) – patients who did not require intensive care.

The primary outcome was PICU admission during the MIS-C episode, used as the dependent variable in logistic regression modeling. Secondary outcomes assessed descriptively included PICU length of stay (days) and total hospital length of stay (days); length-of-stay variables were not used as candidate predictors in model development.

#### 2.1.4 PICU admission criteria and clinical decision-making

PICU admission decisions were made by the on-call pediatric intensivist at the time of initial assessment. Although no single standardized triage score (e.g., Pediatric Early Warning Score [PEWS]) was prospectively mandated and recorded across all sites, admission decisions were primarily driven by objective physiologic instability and/or the need for organ support. Typical indications included: hemodynamic instability or shock requiring vasoactive support and/or advanced fluid resuscitation; respiratory failure or escalating respiratory support (high-flow oxygen, non-invasive or invasive ventilation); altered mental status requiring continuous monitoring; and evolving organ dysfunction requiring intensive monitoring and rapid escalation of therapy. In routine practice, validated pediatric severity/organ dysfunction tools (e.g., Bedside PEWS, pSOFA, PELOD-2, PRISM III) may complement clinical judgment; however, these scores were not uniformly documented and therefore were not used as study variables or formal inclusion/exclusion criteria for PICU admission.

### 2.2 Statistical analysis

The analytical procedures were performed using the statistical software EZR v.1.54. Descriptive statistics for potential prognostic factors were presented for quantitative variables were presented as median and interquartile range (Me (IQR) or as mean ± standard deviation (M ± SD), depending on normality. Normality of data distribution was assessed using the Shapiro–Wilk W-test. Qualitative (binary) variables were expressed as absolute and relative frequencies (n, %). Intergroup comparisons were conducted using the Student's t-test or Mann–Whitney U-test for continuous variables, and the *χ*^2^-test or Fisher's exact test for categorical variables. A two-tailed *p*-value < 0.05 was considered statistically significant.

The following analysis involved univariate logistic regression (ULR), with a *p* < 0.1 defined as the threshold for statistical significance.

Variable selection for the multivariable model was performed using a forward stepwise approach based on the Akaike information criterion (AIC).

We assessed multicollinearity using the variance inflation factor (VIF) and considered values below 5 as acceptable.

To develop severity scoring models for MIS-C, multivariate logistic regression (MLR) was applied, and the independent association of each predictor with the outcome was deemed statistically significant when *p* < 0.05. The predictive ability of the model was evaluated using receiver operating characteristic (ROC) curve analysis. The area under the curve (AUC) was computed to determine the model's discriminative accuracy. Odds ratios (ORs) with corresponding 95% confidence intervals (CIs) were subsequently calculated to estimate the strength of association and to visualize the probability of classification errors. The optimal cut-off point for continuous variables was determined using the Youden index. Specifically, ROC analysis for each continuous biomarker was performed with PICU admission (PICU+ vs. PICU–) as the reference outcome, and the threshold maximizing the Youden index (sensitivity plus specificity minus 1) was selected. The resulting cut-offs were used to dichotomize variables for subsequent regression analyses and score construction (e.g., ESR ≥ 22 mm/h; IL-6 ≥ 310 pg/mL).

Internal validation of the prognostic models was performed using bootstrap resampling with 1,000 iterations. Model performance was evaluated across replications based on the area under the ROC curve (AUC) and the Brier index, and their mean values with 95% confidence intervals were estimated.

For practical implementation of the developed models, two scoring systems were designed: the MIS-C – Severity Score Basic (MIS-C-SS-Basic) for healthcare facilities with limited resources, and the MIS-C – Severity Score Advanced (MIS-C-SS-Advanced) for institutions with enhanced access in laboratory tests, especially to interleukin-6 (IL-6) measurement. Each factor incorporated into the corresponding model was assigned an index value determined by its weighting coefficient (*β*), indicating its relative impact in each model.

## 3 Results

### 3.1 Baseline characteristics of the cohort stratified by PICU admission

The study cohort included 51 children; 24 (47%) required PICU admission. The median PICU length of stay lasted 5.5 (3.75–6.25) days (*p* < 0.001; W = 0.565). Patients admitted to the PICU had a longer total hospital stay, with a statistically significant difference — 15 (11–17) vs. 11 (9–14) days (*p* = 0.011).

The median age of children who required PICU admission was 6.00 (2.00–10.00) years, compared with 6.55 (4.00–10.00) years in those treated without intensive care. In the PICU group, boys predominated, with a male-to-female ratio of 2.4:1 (17 (70%) vs. 7 (29%)). In contrast, the sex ratio was nearly balanced among children who did not require intensive care — 0.9:1 (13 (48%) vs. 14 (52%)).

The sex distribution did not differ significantly between groups (*p* = 0.374 and *p* = 0.154, respectively). A comprehensive baseline description of demographic, clinical, and major laboratory parameters stratified by PICU admission is provided in the Supplementary Table S1 regardless of statistical significance. In the following sections, we focus on clinically relevant differences and key predictors associated with PICU requirement.

### 3.2 Univariable сomparative analysis of clinical characteristics between PICU (+) and PICU (–) groups

Univariable comparisons between PICU+ and PICU− groups are summarized in Tables 1, 2. Table 1 presents baseline clinical characteristics, including ultrasound findings, whereas Table 2 presents baseline laboratory parameters. These analyses provide the descriptive context for subsequent univariable logistic regression assessing associations with PICU admission. Variables with *p* < 0.10 in preliminary comparisons were entered into ULR as a screening step to avoid excluding potentially clinically relevant predictors.

**Table 1:** Clinical characteristics of MIS-C patients by PICU status (PICU+ vs PICU−).

Parameter	Data presentation	PICU (+), *n* = 24	PICU (-), *n* = 27	*p*-value	OR [95% CI]
Comorbidity (≥ 2 nosologies)	n (%)	5 (21)	0 (0)	0.018	15.51 [0.81–297.19]*
Amount of involved systems	n (%)				
4		22 (92)	16 (60)	0.011	7.28 [1.32–76.41]
5		16 (67)	9 (33)	0.025	3.88 [1.08–15.15]
6		8 (33)	1 (4)	0.009	12.41 [1.44–596.44]
Pharyngeal hyperemia	n (%)	17 (71)	11 (41)	0.048	3.44 [0.96–13.50]
Pharyngitis		9 (38)	1 (4)	0.004	14.84 [1.76–706.5]
Cough	n (%)	11 (46)	4 (15)	0.029	4.71 [1.11–24.57]
Cardiovascular changes (CVC):	n (%)	18 (75)	12 (44)	0.045	3.65 [0.99–15.05]
Pericarditis		13 (54)	5 (19)	0.010	5.02 [1.28–22.97]
Sinus tachycardia		12 (50)	3 (11)	0.005	7.65 [1.65–50.36]
CVC without CA-involvement		17 (71)	9 (33)	0.011	4.69 [1.28–19.03]
Hepatomegaly	n (%)	14 (58)	4 (15)	0.001	7.68 [1.83–40.47]
Free fluid in abdominal cavity		12 (50)	6 (22)	0.046	3.41 [0.90–14.26]
Ascites		11 (46)	5 (19)	0.068	3.62 [0.91–16.51]
Meningism	n (%)	10 (42)	3 (11)	0.022	5.51 [1.16–36.46]
Restlessness		8 (33)	3 (11)	0.087	3.89 [0.78–26.24]

*OR and 95% CI were corrected using the Haldane–Anscombe adjustment due to the absence of events in the PICU (–) group; CVC, cardiovascular changes; CA-involvement, coronary arteries involvement.

**Table 2:** Laboratory parameters of MIS-C patients by PICU status (PICU+ vs PICU−).

Parameter	Data presentation	PICU (+), *n* = 24	PICU (-), *n* = 27	*p*-value	OR [95% CI]
WBC, *10^9^/L	Me (IQR)	18.6 (13.5–25.5)	14.7 (11.4–20.9)	0.091	—
ESR, mm/h	M ± SD	37.9 ± 14.9	28.9 ± 14.5	0.040	—
Albumin, g/L	Me (IQR)	30.9 (29.5–32.0)	28.5 (27.0–30.0)	0.002	—
Laboratory evidence of coagulopathy	n (%)	18 (75)	13 (48)	0.084	3.15 [0.85–12.94]
IL-6, pg/mL	Me (IQR)	311.2 (223.3–311.5)	149.6 (149.0–210.5)	0.041	—

ESR, erythrocyte sedimentation rate; IL-6, interleukin 6; WBC, white blood cells.

As shown in Table 1, several baseline clinical characteristics were compared between PICU+ and PICU− groups, allowing identification of clinically relevant differences to be explored in univariate analyses.

#### 3.2.1 Comorbidity

The overall presence of any comorbidity as a potential factor influencing MIS-C severity did not differ significantly between the PICU (+) and PICU (–) groups, being observed in 14 (58%) and 11 (41%) patients (*p* = 0.267). At the same time, as shown in Table 1, the analysis of comorbidity profiles between the groups revealed a statistically significant difference (*p* = 0.018). Children with two or more coexisting conditions were observed more frequently in the PICU (+) group — 5 cases (21%) — while no such cases were recorded among patients who did not require intensive care.

#### 3.2.2 Multisystem involvement

The diagnostic criterion for MIS-C requires the involvement of at least two organ systems; however, statistical significance was observed when four or more systems were affected. Specifically, the analysis demonstrated significant associations for the involvement of 4, 5, and 6 or more systems.

#### 3.2.3 Mucocutaneous involvement

Statistically significant differences in mucosal involvement were observed for two key clinical signs—pharyngeal hyperemia and pharyngitis. Hyperemia of the oropharynx was identified in 71% of patients in the PICU (+) group, compared to 41% in the PICU (−) group (*p* = 0.048; OR = 3.44, 95% CI [0.96–13.50]). CI [1.76–706.5]), highlighting its potential value as a prognostic factor. Given the wide confidence interval and the crossing of the null value in the first case, only pharyngitis was selected for further analysis, due to its clearer statistical robustness and lower risk of error.

#### 3.2.4 Cardiovascular symptoms

Analysis of the cardiovascular involvement block revealed a significant difference in the overall rate of cardiac and vascular involvement (*p* = 0.045, OR = 3.65, 95% CI [0.99–15.05]), which included myocarditis, pericarditis, coronary artery (CA) changes, hypotension, shock, and reduced ejection fraction (EF). Notably, when coronary artery abnormalities were excluded from this composite variable, the difference between groups remained significant (*p* = 0.011, OR = 4.69, 95% CI [1.28–19.03]), and was therefore included in subsequent analysis. Additionally, higher odds ratios were observed in the PICU group for pericarditis (*p* = 0.010, OR = 5.02, 95% CI [1.28–22.97]) and for sinus tachycardia in the absence of hyperthermia (*p* = 0.005, OR = 7.65, 95% CI [1.65–50.36]).

#### 3.2.5 Gastrointestinal manifestation

Among the manifestations of the abdominal syndrome, hepatomegaly (determined by ultrasound examination according to age- and body size-adjusted reference ranges (13) was found to differ significantly between groups. It was observed in 58% of patients admitted to the PICU compared with 15% of those managed outside the PICU (*p* = 0.001, OR = 7.68, 95% CI [1.83–40.47]). The presence of free fluid in the abdominal cavity (detected by ultrasound at any localization) was considered in this study distinctively from ascites, representing a phenomenon with a subclinical course and primarily instrumental verification. The prognostic potential of both findings was regarded as relevant, based on their higher frequency among patients admitted to the PICU compared with those managed outside it — 50% vs. 22% (*p* = 0.046, OR = 3.41, 95% CI [0.90–14.26]) for free fluid and 46% vs. 19% (*p* = 0.068, OR = 3.62, 95% CI [0.91–16.51]) for ascites, respectively. An additional analysis was conducted according to the localization of the detected free fluid within the abdominal cavity. The distribution was as follows: iliac region – 5 (28%), pelvic cavity – 9 (50%), inter-intestinal space – 7 (39%), and perihepatic area – 8 (44%). The median fluid depth, as measured by ultrasound across all sites, was 15 mm (10–20 mm). Among patients with free abdominal fluid, the recorded maximal depth showed wide overlap between groups (PICU+: 17.50 (10.00–20.00) mm vs. PICU–: 12.50 (7.75–15.00) mm; *p* = 0.151), suggesting that small-volume effusions may reflect generalized inflammation and have limited specificity for severe disease. Notably, the smallest documented effusion depth was 7.0 mm.

#### 3.2.6 Respiratory system involvement

Cough was observed more frequently among patients in the PICU (+) group (46% vs. 15%, *p* = 0.029). The odds ratio of 4.71 (95% CI [1.11–24.57]) underscores the need for careful monitoring of respiratory status when assessing the risk of a more severe MIS-C course.

#### 3.2.7 Neurological manifestations

Meningism occurred more often in the PICU (+) group (42% vs. 11%, *p* = 0.022, OR = 5.51, 95% CI [1.16–36.46]). A similar tendency, though with a higher *p*-value (*p* = 0.087), was observed for irritability (33% vs. 11%, OR = 3.89, 95% CI [0.78–26.24]).

#### 3.2.8 Laboratory findings

Baseline laboratory parameters stratified by PICU admission are summarized in Table 2.

Table 2 shows that WBC tended to be higher in the PICU(+) group, although the difference was not statistically significant (*p* = 0.091). PICU(+) patients had higher inflammatory markers, including ESR (37.9 ± 14.9 vs. 28.9 ± 14.5 mm/h; *p* = 0.041) and IL-6 (311.2 [223.3–311.5] vs. 149.6 [149.0–210.5] pg/mL; *p* = 0.041), and lower albumin levels (28.5 [27.0–30.0] vs. 30.9 [29.5–32.0] g/L; *p* = 0.002). Laboratory evidence of coagulopathy was more frequent among PICU(+) patients than PICU(–) patients (75% vs. 48%, *p* = 0.084).

Overall, the laboratory profile in the cohort of patients with severe MIS-C requiring intensive care was characterized by a more pronounced inflammatory response and hypoalbuminemia.

In univariable comparisons, PICU (+) patients differed significantly from PICU (–) patients in comorbidity burden (≥ 2 nosologies), extent of multisystem involvement (4–6 systems), selected clinical (pharyngeal hyperemia, pharyngitis, pericarditis, sinus tachycardia, cardiovascular changes without coronary artery involvement, cough, and meningism)/ultrasound changes (hepatomegaly and free abdominal fluid), and inflammatory markers (ESR, IL-6, and albumin). Variables that differed between groups in univariable comparisons were further evaluated using ULR to screen candidate predictors of PICU admission.

### 3.3 Univariate logistic regression analysis of prognostic factors

To identify prognostic factors, we used a ULR analysis. For patients with MIS-C who required PICU admission (*n* = 24) the dependent variable was defined as Y = 1, and Y = 0 for those managed outside the PICU (*n* = 27). Results of the ULR are shown in Table 3.

**Table 3:** Univariate logistic regression analysis for predictors of severe MIS-C in children.

Prognostic factor	Model coefficient,b ± m	OR [95% CI]	р-value	AUC [95% CI]
Amount of involved systems				
4	2,02 ± 0,84	7.56 [1.47–38.93]	0.016	0.66 [0.55–0.77]
5	1,39 ± 0,60	4.00 [1.25–12.84]	0.020	0.67 [0.53–0.80]
6	1.57 ± 0.74	4.80 [1.12–20.61]	0.035	0.75 [0.62–0.89]
Pharyngeal hyperemia	1,26 ± 0,60	3.53 [1.10–11.40]	0.034	0.65 [0.52–0.79]
Pharyngitis	2,75 ± 1,10	15.60 [1.80–135]	0.013	0.68 [0.56–0.77]
Cough	1,25 ± 0,62	4.87 [1.29–18.40]	0.019	0.69 [0.55–0.83]
Cardiovascular changes (CVC)	1,32 ± 0,61	3.75 [1.13–12.40]	0.030	0.65 [0.52–0.79]
Pericarditis	1,64 ± 0,64	5.20 [1.47–18.30]	0.010	0.68 [0.55–0.80]
Sinus tachycardia	2,08 ± 0,74	8.00 [1.89–33.90]	0.005	0.69 [0.58–0.81]
CVC without CA-involvement	1,58 ± 0,61	4.86 [1.48–16.0]	0.009	0.69 [0.56–0.82]
Hepatomegaly	2,08 ± 0,68	8.05 [2.12–30.60]	0.002	0.72 [0.59–0.84]
Free fluid in abdominal cavity	1,25 ± 0,62	3.50 [1.04–11.70]	0.042	0.64 [0.51–0.77]
Meningism	1,58 ± 0,68	5.71 [1.34–24.30]	0.018	0.70 [0.56–0.84]
ESR, mm/h (≥ 22,00)	1,11 ± 0,63	3.04 [0.88–10.50]	0.078	0.62 [0.49–0.75]
Albumin, g/l (< 32,00)	−2,37 ± 1,11	0.09 [0.01–0.83]	0.033	0.63 [0.53–0.73]
IL-6, pg/mL (≥ 310,00)	2,77 ± 0,75	16.00 [3.68–69.60]	< 0.001	0.78 [0.66–0.89]

As shown in Table 3, ULR models identified several clinical and laboratory parameters that were significantly associated with the likelihood of a severe course of MIS-C. A higher risk of severe MIS-C was observed in patients presenting with involvement of six organ systems (*p* = 0.035, OR = 4.80, 95% CI [1.12–20.61]), hepatomegaly (*p* = 0.002, OR = 8.05, 95% CI [2.12–30.60]), and elevated IL-6 levels exceeding 310 pg/mL (*p* < 0.001, OR = 16, 95% CI [3.68–69.60]). Clinically significant predictors also included pharyngitis (*p* = 0.013, OR = 15.60, 95% CI [1.80–135.00]) and the presence of free fluid in the abdominal cavity (*p* = 0.040, OR = 5.80, 95% CI [1.10–11.70]). Hypoalbuminemia (< 32.0 g/L) was associated with an increased risk of severe disease (*p* = 0.033, OR = 0.09, 95% CI [0.01–0.83]), indicating its potential diagnostic value.

Thus, the results indicate that multisystem involvement, combined with pronounced inflammatory activity and hepatomegaly, represents an important predictor of adverse disease course in MIS-C and may be integrated into risk stratification models.

### 3.4 Prognostic models of MIS-C severity adapted to the resource settings of healthcare facilities

The MLR model was developed based on variables that demonstrated discriminatory ability in predicting the risk of intensive care unit admission and the overall severity of MIS-C.

**Figure 1:**
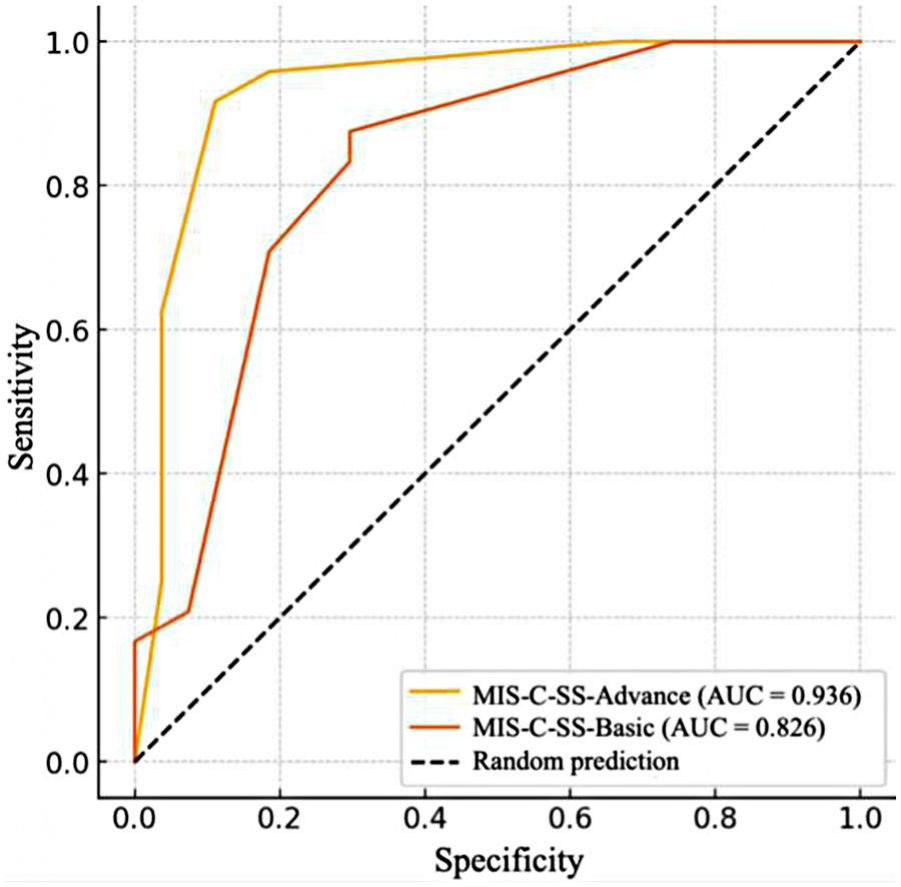
Comparative ROC curves for the MIS-C-SS-basic and MIS-C-SS-advanced models.

Considering the need for tiered stratification according to the level of healthcare resources, two operational models were developed. The Advanced model included involvement of four organ systems, hepatomegaly, and interleukin-6 (IL-6) levels above 310.0 pg/mL, designed for centers with access to routine and rapid IL-6 testing. In contrast, the Basic model is proposed for institutions without such access and comprises the presence of free abdominal fluid (excluding ascites), hepatomegaly, and a dichotomous ESR variable (threshold ≥ 22 mm/h). Both models demonstrated a high degree of internal stability, with all included predictors retaining statistical significance (*p* < 0.05). A comparative visualization of the two resulting models is presented in Figure 1.

Figure 1 presents two ROC curves that markedly deviate from the line of random classification (AUC = 0.5), indicating good discriminative ability of both models. It should be noted that the stability of the prognostic model for MIS-C severity was strongly influenced by the laboratory marker included in it. Incorporation of interleukin-6 increased the AUC value to 0.936 (95% CI [0.86–1.00]), whereas substitution with ESR reduced it to 0.826 (95% CI [0.71–0.94]). The summarized odds ratios and 95% confidence intervals for both models are presented in Table 4.

**Table 4:** Statistical parameters of multivariate logistic regression models.

Indicator	MIS-C-SS-Advanced model		MIS-C-SS-Вasic model	
Sensitivity, %	91,7		87.5	
Specificity, %	88,9		70.4	
Оptimal сut-off	0.68		0.31	
Predictor variable	OR [95% CI]	*p*-value	OR [95% CI]	*p*-value
Four system involvement	19.10 [1.27–288.00]	0.033	—	—
Free fluid in abdominal cavity	—	—-	9.05 [1.56–52.70]	0.014
Hepatomegaly	14.50 [2.12–98.60]	0.006	8.29 [1.89–36.40]	0.005
IL-6	63.50 [5.49–733.00]	<0.001	—	—
ESR	—	—	6.63 [1.07–41.20]	0.043

Table 4 presents the results of the multivariate logistic regression analysis for two predictive models of MIS-C severity — MIS-C-SS-Advanced and MIS-C-SS-Basic.

Both models demonstrated significant associations between clinical and laboratory predictors. In the MIS-C-SS-Advanced model, the most powerful predictor was interleukin-6 (IL-6) level (*p* < 0.001, OR = 63.5, 95% CI [5.5–733.0]), followed by involvement of four organ systems (*p* = 0.033, OR = 19.1, 95% CI [1.3–288.0]) and hepatomegaly detected by ultrasound (*p* = 0.006, OR = 14.5, 95% CI [2.1–98.6]). In the MIS-C-SS-Basic model, which did not include IL-6, all three predictors remained statistically significant — hepatomegaly (*p* = 0.005, OR = 8.3, 95% CI [1.9–36.4]), presence of free abdominal fluid (*p* = 0.014, OR = 9.1, 95% CI [1.6–52.7]), and elevated ESR (*p* = 0.043, OR = 6.6, 95% CI [1.1–41.2]).

The MIS-C-SS-Advanced model demonstrated the highest discriminatory ability (AUC = 0.936). An optimal cut-off value of 0.68 provided high sensitivity (92%), allowing the detection of nearly all severe cases, with a specificity of 89%. Given these parameters, the model is unlikely to miss patients who truly require intensive care, although a small proportion of children with moderately severe disease may be classified as false positives. However, this characteristic is particularly valuable in clinical settings, where the primary priority is to avoid missing critically ill patients.

The MIS-C-SS-Basic model, which incorporated ESR instead of interleukin-6, showed a slightly lower yet still clinically meaningful predictive accuracy (AUC = 0.826, cut-off = 0.31, sensitivity = 87.5%, specificity = 70.4%).

The Basic model showed lower discriminative capacity than the Advanced version; nevertheless, its use remains justified in healthcare facilities with limited resources, where IL-6 testing is either unavailable or time-consuming and therefore not clinically relevant in critical conditions requiring urgent decision-making and patient triage.

Thus, both models can be regarded as reliable tools for predicting the risk of severe MIS-C. However, the Advanced model should be prioritized in centers with access to comprehensive laboratory testing, while the Basic model represents a practical adaptation for resource-limited settings, ensuring a balance between diagnostic accuracy and accessibility.

### 3.5 Internal validation

**Figure 2:**
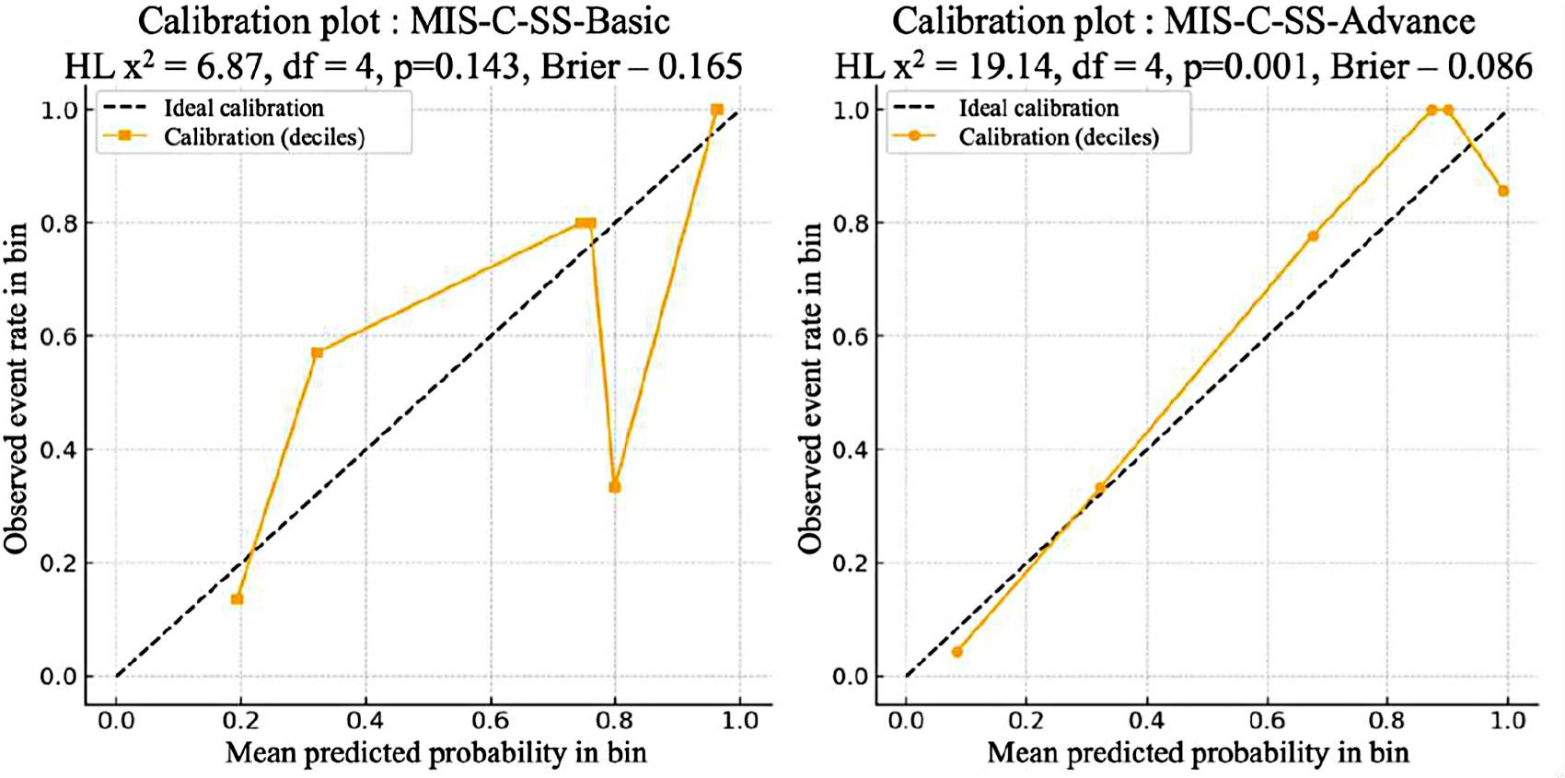
Calibration curves of the predictive models MIS-C-SS-basic (left) and MIS-C-SS-advanced (right) obtained using the bootstrap resampling method.

Considering the relative sample size (*n* = 51), internal validation of the predictive models was performed using the bootstrap resampling method (bootstrap validation, BV) with B = 1,000 iterations. To assess model performance, the bias-corrected area under the ROC curve (AUC) was estimated, calibration plots were constructed, and the Brier score was calculated. Visualization of the bootstrap-calibrated plots is presented in Figure 2.

The results of the bootstrap validation are summarized in Table 5.

**Table 5:** Results of internal validation of MIS-C severity prediction models.

Model	AUC [95% СI]	Brier score	HL *χ*^2^(df)	HL *p*-value
MIS-C-SS-Advance	0.936 [0.86–0.99]	0.086	19.14 (4)	0.001
MIS-C-SS- Basic	0.826 [0.74–0.94]	0.165	6.87 (4)	0.143

HL χ^2^, Hosmer–Lemeshow test; df, degree of freedom.

Table 5 summarizes the main statistical results of both models after bootstrap validation with 1,000 iterations. The comparative analysis demonstrates good overall predictive performance of both models, though with differing levels of accuracy.

The Advanced model exhibited enhanced performance in terms of its discrimination capacity (AUC = 0.94 [0.86–0.99]) and overall accuracy (lower Brier score = 0.086). However, the Hosmer–Lemeshow test on the development dataset revealed evidence of imperfect group calibration (*p* = 0.001), which should be carefully considered during external validation. In comparison, the Basic model retained an acceptable level of predictive validity (higher Brier score = 0.165, but HL *p* = 0.143), indicating adequate calibration across risk deciles, which is particularly relevant in settings with restricted access to laboratory diagnostics. Hence, both models may be implemented in routine clinical practice, although the Advanced model is preferable when interleukin-6 measurement is available.

### 3.6 Development of a prognostic scoring system for MIS-C severity assessment

To facilitate clinical implementation by physicians, both Advanced and Basic logistic regression models were transformed into point-based scoring systems. This approach serves as an optimization strategy to enhance simplicity and speed of use in routine and emergency clinical settings.

**Figure 3:**
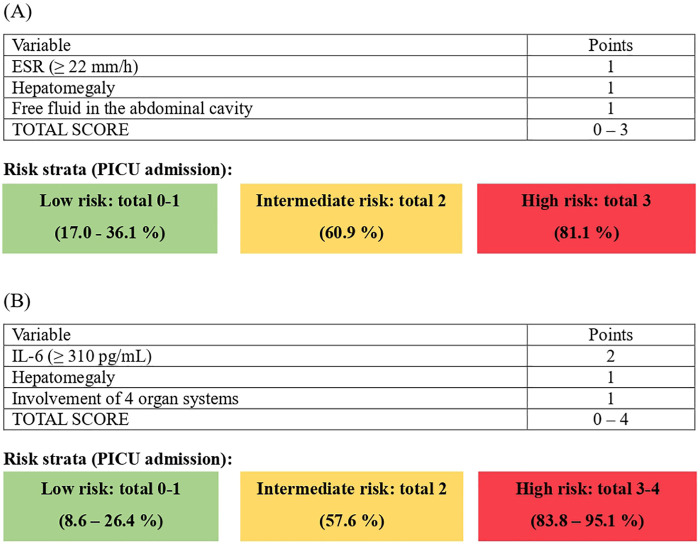
MIS-C severity scoring sheets with color-coded risk strata for PICU admission. **(A)** MIS-C-SS-Basic. **(B)** MIS-C-SS-Advanced.

Each predictor variable with independent prognostic value was assigned a specific score using the relative *β*-coefficient method. The smallest absolute *β*-coefficient within each final model was taken as the reference unit (Basic: ESR, *β* = 1.89; Advanced: hepatomegaly, *β* = 2.67), and all other coefficients were scaled and rounded proportionally to the nearest integer. The reference predictor (|*β*|) within each model was set to 1 point. Points for the remaining predictors were obtained by dividing each *β*-coefficient by this reference value and rounding the result to the nearest integer. Detailed scoring system and risk strata are presented in Figure 3.

As shown in Figure 3, the MIS-C-SS-Basic model included three predictors: ESR ≥ 22 mm/h, hepatomegaly, and the presence of free fluid in the abdominal cavity, each assigned 1 point. The MIS-C-SS-Advanced model comprised IL-6 ≥ 310 pg/mL (2 points), involvement of four organ systems, and hepatomegaly (1 point each). IL-6 was assigned 2 points because it had the largest regression coefficient in the Advanced model (*β* = 4.15) within the relative *β*-coefficient approach.

To define low-, intermediate-, and high-risk categories, each possible total score was mapped to the corresponding predicted probability of PICU admission derived from the underlying logistic regression model (using the model intercept and coefficients). Based on the monotonic increase in predicted risk across point totals and to provide three clinically pragmatic triage strata, cut-offs were defined as follows: for MIS-C-SS-Basic (0–3 points), low risk = total 0–1 (= 17.0–36.1%), intermediate risk = total 2 (= 60.9%), and high risk = total 3 (= 81.1%); for MIS-C-SS-Advanced (0–4 points), low risk = total 0–1 (= 8.6–26.4%), intermediate risk = total 2 (= 57.6%), and high risk = total 3–4 (= 83.8–95.1%). These thresholds are shown in Figure 3.

The optimal cut-off value for the Basic model was 2 points, providing a sensitivity of 95.8% and specificity of 81.5%. For the model including IL-6, the optimal threshold was 3 points, yielding a sensitivity of 87.5% and specificity of 92.6%.

Additionally, both scoring systems were used to calculate the percentage risk of severe MIS-C and the need for intensive care unit admission. The probabilities were derived using the logistic equation, constructed from the intercept (*β*0) and the smallest significant *β*-coefficient as the step per point. The data presented in Figure 3 demonstrate a direct relationship between the total score and the predicted risk of severe MIS-C. According to the results, each patient has a non-zero baseline probability of severe disease within the applied model, even in the absence of any risk factor – 17.0% for MIS-C-SS-Basic and 8.6% for MIS-C-SS-Advance.

As the total score increases, the predicted risk rises accordingly. At 2 points, the probability exceeds the 50% threshold for both models (60.9% for Basic and 57.6% for Advance). At 3 points, the risk of severe disease reaches 81.1% in the Basic model and 83.8% in the Advanced model. The maximum score of 4 points corresponds to the highest predicted risk of 95.1%.

**Figure 4:**
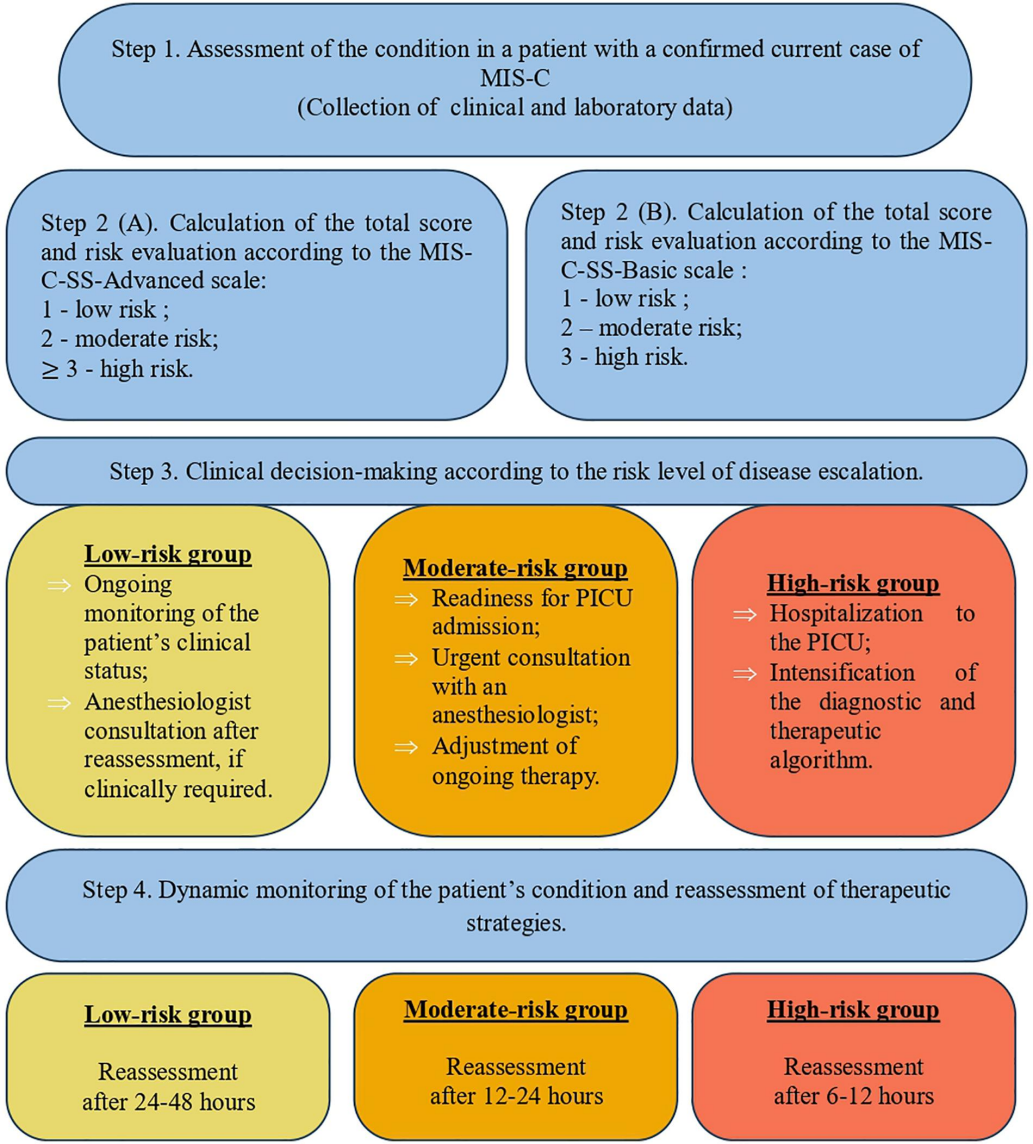
Stepwise algorithm for clinical application of MIS-C-SS-Advanced and MIS-C-SS-Basic in assessing risk of severe MIS-C and PICU admission.

Considering the characteristics of the laboratory parameters included in the developed scoring systems – interleukin-6 as a more specific but less accessible biomarker, and ESR as a less specific marker with slower dynamic changes—it appears reasonable to apply a dynamic use of the scoring approach for appropriate patient management. Figure 4 illustrates the proposed algorithm for implementing the scoring systems and the corresponding clinical decision-making pathway.

The Figure 4 illustrates a stepwise approach for implementing the MIS-C-SS-Advanced and MIS-C-SS-Basic scoring systems in clinical practice. The algorithm comprises four sequential stages: initial patient assessment, risk stratification into three categories, with preliminary branching depending on availability of laboratory diagnostics, followed by subsequent clinical management options.

Understanding the percentage risk of requiring intensive care enables the use of these scoring systems as a tool for guiding clinical and strategic decision-making in patient triage. The Basic model can be applied in any healthcare setting; however, its lower discriminative ability should be considered, implying a higher likelihood of false-positive or false-negative assessments. In contrast, incorporating interleukin-6 levels into the evaluation enhances the accuracy and reliability of clinical decision-making.

The proposed scoring systems allow for a rapid assessment of the risk of PICU admission in patients with MIS-C. Their high sensitivity with a minimal number of available variables makes them suitable for initial patient triage in resource-limited clinical settings. In cases where IL-6 measurement is available, the Advanced version of the score provides greater discriminative performance.

## 4 Discussion

The developed prognostic scoring systems for MIS-C following SARS-CoV-2 exposure provide a clinically relevant tool that is statistically robust and adaptable across healthcare settings of varying resource availability.

In modern pediatric practice, scoring systems such as PRISM III, PELOD-2, and the pediatric-adapted SOFA (pSOFA) are commonly used. These tools standardize the assessment of severity in urgent pediatric patients, which has prompted attempts to adapt them for children with MIS. However, none of these scales have been specifically designed for the stratification of patients with MIS-C.

For instance, PRISM III demonstrates a high predictive accuracy for patient mortality and has undergone long-term international validation. However, in clinical scenarios that require immediate patient triage and rapid referral, particularly under resource-constrained conditions, its dependence on a broad array of laboratory parameters may represent a practical limitation for diagnostic use (14–16). Emphasizing of PELOD-2 score on multisystem involvement and the option for serial dynamic monitoring, offers a clear methodological advantage. Nevertheless, its high resource demands and the lack of parameter specificity for MIS-C limit its utility in low- and middle-income country (LMIC) settings (17, 18). The pSOFA scale, which is oriented toward organ dysfunction and based on the assessment of six organ systems, does not require extensive laboratory testing and is routinely applied in emergency and intensive care settings, which aligns with the operational context of our proposed models (19, 20).

Direct comparison between our MIS-C-specific models and general pediatric severity scores may be somewhat biased and non-specific; nevertheless, this contrast underscores the need for validated scoring systems tailored specifically to the stratification of MIS-C patients.

The scoring systems developed in our study – MIS-C-SS-Basic and MIS-C-SS-Advanced – were constructed by integrating both clinical features (hepatomegaly, presence of free abdominal fluid, and involvement of four organ systems) and laboratory characteristics of MIS-C (IL-6, ESR).

In the Advanced model, the presence of the highly specialized laboratory marker interleukin-6 is a key component. This cytokine serves not only as the most statistically significant variable but also as a predictor characterized by high specificity within the scale due to its pathophysiological role in amplifying the inflammatory response during MIS-C. Previous studies have consistently demonstrated that elevated IL-6 levels are strongly associated with severe MIS-C, multi-organ involvement, and the need for intensive care, supporting its inclusion as a robust biomarker in predictive models (21–23).

Despite its absence in the Basic model, which instead incorporated the more accessible ESR, this scale also demonstrated good predictive performance (AUC = 0.826). ESR, as a marker of systemic inflammation, has likewise been reported as a valuable yet pragmatic indicator of disease activity in resource-limited settings (24, 25). The combination of simple calculation methodology, the absence of complex algorithms, and the discriminative ability of both models, while considering the available resources of healthcare facilities, make these scales practically valuable and convenient for implementation as primary triage tools. Nevertheless, certain methodological aspects should be acknowledged when interpreting these results. This study has several limitations that should be considered when interpreting the findings. The limited sample size (*n* = 51) from a single geographical region constrains the precision and generalisability of the results and may predispose the models to overfitting and optimistic performance estimates despite bootstrap internal validation. In addition, the primary outcome (PICU admission) may be influenced by institutional practices (triage thresholds, bed availability, staffing, and local resource constraints). While admission decisions were guided by objective clinical instability and organ support requirements assessed by pediatric intensivists at presentation, a single standardized early warning score (e.g., PEWS) was not uniformly implemented or documented across sites. Therefore, outcome misclassification due to practice variability cannot be excluded and should be considered when interpreting model performance. External validation in settings with standardized triage criteria is warranted. To strengthen the relevance and applicability of the proposed scoring systems, external validation in independent cohorts is a crucial next step.

Ultrasound findings were obtained retrospectively from routine clinical reports performed by different operators; therefore, inter-observer variability and limited standardization could not be fully controlled. In addition, small-volume serous effusions may reflect systemic inflammation and have limited specificity, which should be considered when interpreting their prognostic contribution.

## 5 Conclusions

The proposed scoring systems are statistically validated tools for predicting both the risk of intensive care unit admission and the overall severity of MIS-C in the pediatric population. Their adaptation to healthcare settings with varying levels of resource availability was based on the high accuracy of the MIS-C-SS-Advanced model, which includes interleukin-6, and on the acceptable predictive performance – but greater clinical accessibility – of the MIS-C-SS-Basic model, which relies primarily on clinical and routine laboratory parameters.

## Data Availability

The raw data supporting the conclusions of this article will be made available by the authors, upon reasonable request and without undue reservation.
