# Supplementary material for: Prognostic models of multisystem inflammatory syndrome severity according to resource availability in healthcare facilities in Ukraine
Source: Front Pediatr. 2026 Feb 26;14:1740828. doi: 10.3389/fped.2026.1740828 (PMC12979511; doi:10.3389/fped.2026.1740828)
Supplement: Supplementary file 1 [file Table1.docx]

Supplementary Table 1. Baseline descriptive characteristics of the MIS-C cohort overall and stratified by PICU admission (PICU+ vs PICU−)

| Variables | Total (N = 51) | PICU (+) (n=24) | PICU (-) (n=27) | p-value |
| --- | --- | --- | --- | --- |
| Recruitment timeframe, MM/DD/YYYY | 01/10/2020–10/31/2022 | 01/10/2020–10/31/2022 | 01/12/2020–07/09/2022 | --- |
| Hospital LOS, days | 12  (10 – 16) | 15  (11–17) | 11  (9–14) | 0.011 |
| PICU LOS, days | 0 (0 –5) | 5.5  (11 –17) | 0 | < 0.001 |
| Age, year | 6.10  (3.00 – 10.00) | 6.55  (4.00 – 10.00) | 6.00  (2.00 – 10.00) | 0.374 |
| Sex, n (%)  Male  Female | 30 (58.82)  21 (41.18) | 17 (70)  7 (29.17) | 7 (29)  14 (51.85) | 0.154 |
| Any comorbidity, n (%) | 25 (49.02) | 14 (58.33) | 11 (40.74) | 0.267 |
| Comorbidity, n (%)  (≥ 2 nosologies) | 5 (9.80) | 5 (21) | 0 (0) | 0.018 |
| Сomorbidity profile, n (%):  Obesity  CVS disease  Renal abnormality  Chronic lung disease  Gastrointestinal disease  Epilepsy | 6 (11.76)  5 (9.80)  11 (21.57)  2 (3.92)  2 (3.92)  1 (1.96) | 4 (16.67)  3 (12.50)  8 (33.33)  2 (8.33)  2 (8.33)  0 (0) | 2 (7.41)  2 (7.41)  2 (11.11)  0 (0)  0 (0)  1 (3.7) | 0.402  0.656  0.088  0.216  0.216  0.999 |
| Vaccination status (documented*), n (%) | 22 (43.14) | 16 (66.67) | 6 (22.22) | 0.541 |
| System involvement, n (%):  Mucocutaneous involvement  Lymphadenopathy  Respiratory involvement  Cardiovascular involvement  Gastrointestinal involvement  Neurological involvement  Renal involvement  Hematologic involvement | 48 (94.12)  23 (45.10)  33 (64.71)  30 (58.82)  33 (64.71)  35 (68.63)  12 (23.53)  34 (66.67) | 22 (91.67)  13 (54.17)  17 (70.83)  18 (75.00)  18 (75.00)  17 (70.83)  7 (29.17)  18 (75.00) | 26 (96.30)  10 (37.04)  16 (59.26)  12 (44.44)  15 (55.56)  18 (66.67)  5 (18.52)  16 (59.26) | 0.595  0.267  0.558  0.045  0.240  0.772  0.511  0.372 |
| Laboratory parameters:  WBC, x 10*9/L  ANC, x 10*9/L  ALC, x 10*9/L  Hemoglobin, g/L  Platelets, x 10*9/L  ESR, mm/h  Laboratory evidence of coagulopathy (overall)  DD, ng/mL  FBG, g/L  CRP, mg/L  Ferritin, ng/mL  PCT, ng/mL | 16.7 (11.7–22.5)  12.5 (9.4–20.1)  1.5 (0.85–2.5)  113 (103–124)  195 (149–262)  33.2 ± 15.7  31 (60.78)  1760 (840–4080)  5.5 (4.2–7.1)  58.7 (24.5–192)  328 (169–676)  2.1 (0.54–6.9) | 18.6 (13.5–25.5)  14.5 (10.4–23.6)  1.1 (0.83–2.54)  110 (100–121)  181 (145–244)  37.9 ± 14.9  18 (75.00)  2460(1260–5280)  6.2 (4.8–7.5)  120 (49.2–190)  415 (265–840)  3.47 (1.08–9.35) | 14.7 (11.4–20.9)  10.7 (9.2–17.9)  1.8 (1.25–2.49)  114 (104–128)  209 (149–281)  37.9 ± 14.9  13 (48.15)  1240 (620–2920)  5.1 (3.9–6.6)  49.2 (15.9–128.9)  237 (142–500)  1.35 (0.43–5.08) | 0.091  0.174  0.167  0.559  0.358  0.040  0.084  0.143  0.208  0.245  0.108  0.169 |
| Mortality, n (%) | 0 (0) | 0 (0) | 0 (0) | --- |

Note. LOS - length of stay, * - data were N/A for 29 (56.86%) patients, WBC - white blood cells, ANC - absolute neutrophil count, ALC - absolute lymphocyte count, DD - D-dimer (normal < 250), FBG - fibrinogen (normal 2-4), C-reactive protein - (normal < 5.0), PCT - procalcitonin (normal < 0.5).
